# Lipid levels, insulin resistance and cardiovascular risk over 96 weeks of antiretroviral therapy: a randomised controlled trial comparing low-dose stavudine and tenofovir

**DOI:** 10.1186/s12977-018-0460-z

**Published:** 2018-12-14

**Authors:** Alinda G. Vos, Matthew F. Chersich, Kerstin Klipstein-Grobusch, Peter Zuithoff, Michelle A. Moorhouse, Samanta T. Lalla-Edward, Andrew Kambugu, N. Kumarasamy, Diederick E. Grobbee, Roos E. Barth, Willem D. Venter

**Affiliations:** 1 Wits Reproductive Health and HIV Institute, Faculty of Health Sciences, University of Witwatersrand, Johannesburg, South Africa; 2 Julius Global Health, Julius Center for Health Sciences and Primary Care, University Medical Center Utrecht, Utrecht University, Utrecht, The Netherlands; 3 Department of Internal Medicine and Infectious Diseases, University Medical Center Utrecht, Utrecht University, Utrecht, The Netherlands; 4 Division of Epidemiology and Biostatistics, School of Public Health, Faculty of Health Sciences, University of the Witwatersrand, Johannesburg, South Africa; 5 Infectious Diseases Institute, Makerere University, Kampala, Uganda; 6 YRGCARE Medical Centre, CART Clinical Research Site, Voluntary Health Services, Chennai, India

**Keywords:** HIV, South Africa, Uganda, India, Stavudine, Tenofovir, Cardiovascular disease risk

## Abstract

**Background:**

HIV infection and antiretroviral treatment are associated with changes in lipid levels, insulin resistance and risk of cardiovascular disease (CVD). We investigated these changes in the first 96 weeks of treatment with low-dose stavudine or tenofovir regimens.

**Methods:**

This is a secondary analysis of a double blind, randomised controlled trial performed in South-Africa, Uganda and India comparing low-dose stavudine (20 mg twice daily) with tenofovir in combination with efavirenz and lamivudine in antiretroviral-naïve adults (*n* = 1067) (Clinicaltrials.gov, NCT02670772). Over 96 weeks, data were collected on fasting lipids, glucose and insulin. Insulin resistance was assessed with the HOMA-IR index and 10-year CVD risk with the Framingham risk score (FRS). A generalized linear mixed model was used to estimate trends over time.

**Results:**

Participants were on average 35.3 years old, 57.6% female and 91.8% Black African. All lipid levels increased following treatment initiation, with the sharpest increase in the first 24 weeks of treatment. The increase in all lipid subcomponents over 96 weeks was higher among those in the stavudine than the tenofovir group. Insulin resistance increased steadily with no difference detected between study groups. FRS rose from 1.90% (1.84–1.98%) at baseline to 2.06 (1.98–2.15%) at week 96 for the total group, with no difference between treatment arms (*p* = 0.144). Lipid changes were more marked in Indian than African participants.

**Conclusion:**

Lipid levels increased in both groups, with low-dose stavudine resulting in a worse lipid profile compared to tenofovir. Insulin resistance increased, with no difference between regimens. CVD risk increased over time and tended to increase more in the group on stavudine. The low CVD risk across both arms argues against routine lipid and glucose monitoring in the absence of other CVD risk factors. In high risk patients, monitoring may only be appropriate at least a year after treatment initiation.

**Electronic supplementary material:**

The online version of this article (10.1186/s12977-018-0460-z) contains supplementary material, which is available to authorized users.

## Introduction

Globally, cardiovascular disease (CVD) is the leading cause of mortality [1]. Low- and middle-income countries (LMICs) share this burden: the leading cause of death has changed from infectious diseases to ischaemic heart disease over the last two decades [1]. Timely recognition and treatment of cardiovascular risk factors are key for reducing the burden of CVD. Human immunodeficiency virus (HIV) infection and treatment with antiretroviral therapy (ART) affects risk factors for CVD [2–5] and some studies indicate that HIV infection increases the risk of myocardial infarction or stroke by up to 50% [6, 7].

The contribution of ART to CVD risk is less clear, and risk profiles vary by antiretroviral drug [8]. Protease inhibitors and efavirenz, a non-nucleoside reverse transcriptase inhibitor, are well known for their adverse effects on lipid and glucose metabolism [9–11]. Stavudine, a nucleoside reverse transcriptase inhibitor (NRTI), has considerable metabolic side effects. It is associated with mitochondrial toxicity, resulting in lipodystrophy, disturbances in lipid levels and an increase in insulin resistance [12–15]. Stavudine was part of first-line ART for many years globally, as it was well tolerated in the first few months of treatment, cheap, widely available in twice-daily fixed-dose formulations and effective in suppressing viral load [16]. When the extent of mitochondrial toxicity became apparent, use of the drug declined rapidly. Between 2012 and 2016, a clinical trial was initiated, comparing a lower dose of stavudine to the current most commonly used first-line drug, tenofovir disoproxil fumarate (’tenofovir’). The overall conclusion was that low-dose stavudine was equally effective as tenofovir in reducing viral load after 48 weeks, but that lipoatrophy occurred more often in the low-dose stavudine group [17]. Extensive metabolic and toxicity monitoring allow us to conduct an in-depth analysis of the effects of ART initiation with low-dose stavudine or tenofovir on lipid levels, insulin resistance and CVD risk, an important analysis as the vast majority of people on ART are taking regimens containing tenofovir. We present these results in this paper.

## Methods

A randomised 1:1 double blind placebo-controlled trial was conducted in Johannesburg, South Africa, Kampala, Uganda and Chennai, India to assess the efficacy and safety of treatment with either low dose stavudine (20 mg twice a day) or tenofovir (300 mg daily) tablets administered in combination with lamivudine (150 mg BD) and efavirenz (600 mg daily) over 96 weeks (Clinicaltrials.gov, NCT02670772). The methods including quality control and safety evaluation are described in detail elsewhere [17]. In brief, data were captured in an electronic data system. Computerised and manual procedures as well as regular site visits were implemented to optimise data quality. Monitoring and support was undertaken by Pharmaceutical Product Development (PPD, Wilmington, USA). 1067 HIV-positive, ART-naive participants aged 18 years and over with unsuppressed HIV viral load were included from clinical trial sites. Recruitment in India was stopped early due to a regulatory change, and it was decided to raise the number of participants recruited in the remaining sites so as to reach the target number timeously. Exclusion criteria were age above 65 years for the Indian site, pregnancy, CD4+ > 350 cells/μL, hepatitis B antigen positivity, or an estimated glomerular filtration rate (eGFR) < 60 mL/min, calculated using the Cockcroft-Gault equation. Visits took place at baseline, 1 month, 3 months and every 3 months thereafter until 96 weeks. Data collected at baseline included demographic information, medical history including use of medication. In the South African site only, additional data on employment, marital status, having children, as well as data on current smoking, alcohol and drug use were collected.

At each visit, weight, heart rate and blood pressure were measured and blood samples taken. Blood pressure was measured once in a seated position after a 5-min rest. Blood sampling was performed after an overnight fast and included lipids, glucose, insulin, HIV viral load and CD4+ count. Blood samples were analysed by contracted and accredited laboratory services (see Additional file 1: Table S1 for more details). Insulin resistance was quantified with the HOMA-IR, using the following formula: fasting glucose (mmol/L) * fasting insulin (mU/L))/22.5 [18]. Framingham risk score (FRS) was calculated using the 10-year CVD risk score equation, and only available for South Africa, as the risk score includes smoking history [19]. The study was approved by the Human Ethics Research Committee of the University of the Witwatersrand, ethics clearance number 111112, the Research and Ethics Committee of the Joint Clinical Research Centre, Uganda, and the Uganda National Council for Science and Technology clearance number HS 1219, and the IRB & Ethics Research Committee of YRGCARE. All participants provided written informed consent prior to participation. All participants in the stavudine arm were switched to a tenofovir containing regimen at the end of follow as per the national HIV treatment guidelines.

### Statistical analysis

Demographics were reported as means and standard deviations, or medians with interquartile ranges as appropriate. Non-normal outcomes were log transformed to obtain a normal distribution. In line with logistic regression models, we used a logit transformation for the predicted probabilities based on the Framingham risk model. Lipid levels, glucose, insulin, HOMA-IR and FRS measurements over the 96 week period were analysed with a linear mixed model (estimated with restricted maximum likelihood) with a random intercept and a random effect for time. We included a quadratic term for time (i.e. time^2^), considering that outcomes are expected to level off at a certain point in time. Treatment arm, time, time^2^ and the interaction between treatment arm and both time and time^2^ were included as fixed effects together with a correction for age, sex, site of inclusion (South-Africa, Uganda or India), body mass index and viral load at baseline. *p* values for the interactions between treatment and both time and time^2^ were estimated with likelihood ratio tests. Likelihood ratio tests were chosen, as it allows estimation of one *p* value (per outcome) for both interactions. For these tests, models were refitted with maximum likelihood estimation [20]. The estimated mean values at week 0, 24 weeks, 48 weeks, 72 weeks and 96 weeks for all outcomes were displayed graphically (transformed back to the original scale when applicable). The trend over time (per month) per treatment arm was calculated for all outcomes. Results are presented as regression coefficients (β) with 95% confidence intervals. Statistical analysis was done with SPSS version 24 (IBM SPSS Statistics for Windows, Version 24.0. Armonk, NY: IBM Corp.). Validity of the models was evaluated with residual analyses with SAS software version 9.4 (SAS Institute Inc., Cary, NC, USA).

## Results

The mean age of participants was 35.3 years, the majority were female (57.6%), 24.2% was married, 76.0% was employed and most were of African descent (91.8%). The median nadir CD4+ cell count was 206 cells/μL (IQR 124–272). There were no differences in demographics, CVD risk factors or HIV-related factors between the study arms or study sites at baseline (Table 1). Participant retention was similar in each arm, with an overall median follow-up time of 95.4 weeks (Interquartile range (IQR) 95.0–95.7 weeks). Viral suppression (< 50 copies/mL) was reached in 71.1% in the stavudine arm and 76.7% in the tenofovir arm at the end of follow up. In total, 12.2% had a follow-up time of less than 48 weeks. Alcohol use was the most frequently reported substance used, followed by smoking and illegal drug use.

**Table 1:** Demographics, HIV disease and cardiovascular disease risks of patients at baseline

	All (*n* = 1067)	Stavudine (*n* = 533)	Tenofovir (*n* = 534)
Sex, female (*n*, %)	615 (57.6)	324 (60.8)	291 (54.5)
Age in years	35.3 (8.2)	35.5 (8.4)	35.0 (8.1)
Race (*n*, %)			
African	979 (91.8)	489 (91.7)	490 (91.8)
Indian	86 (8.1)	43 (8.1)	43 (8.1)
Other	2 (0.2)	1 (0.2)	1 (0.2)
Site (*n*, %)			
South Africa	600 (56.2)	300 (56.3)	300 (56.2)
Uganda	381 (35.7)	190 (35.6)	191 (35.8)
India	86 (8.1)	43 (8.1)	43 (8.1)
Married^a^ (*n*, %)	139 (24.2)	69 (24.1)	70 (24.2)
Having children^a^ (*n*, %)	492 (85.9)	243 (84.1)	249 (87.7)
Employed^a^ (*n*, %)	434 (76.0)	220 (76.1)	214 (75.9)
CD4+ cell count (cells/µL) (median, IQR)	206 (124–272)	206 (128–274)	206 (123–270)
Log HIV viral load (copies/mL)	11.26 (1.61)	11.34 (1.64)	11.18 (1.58)
History of hypertension^b^ (*n*, %)	59 (5.5)	28 (5.3)	31 (5.8)
History of diabetes mellitus^b^ (*n*, %)	6 (0.6)	4 (0.8)	2 (0.4)
History of dyslipidemia^b^ (*n*, %)	2 (0.2)	1 (0.2)	1 (0.2)
Current smoking^c^ (*n*, %)	86 (15)	44 (15.3)	42 (14.7)
Current alcohol use^c^ (*n*, %)	203 (35.4)	101 (35.1)	102 (35.8)
Current drug use^c^ (*n*, %)	17 (3.0)	8 (2.8)	9 (3.2)
Systolic blood pressure (mmHg)	118.3 (13.2)	117.5 (13.5)	119.1 (12.8)
Diastolic blood pressure (mmHg)	72.9 (10.2)	72.5 (10.6)	73.4 (9.9)
Body mass index (kg/m^2^)	23.5 (4.4)	23.6 (4.6)	23.5 (4.2)
Follow-up time, median months, IQR	95.4 (95.0–95.7)	95.4 (95.0–95.7)	95.4 (95.1–95.8)
Total-C (mmol/L)	4.30 (1.02)	4.39 (1.11)	4.22 (0.93)
HDL-C (mmol/L)	1.32 (0.49)	1.37 (0.54)	1.27 (0.44)
LDL-C (mmol/L)	2.47 (0.86)	2.50 (0.93)	2.44 (0.79)
TG (mmol/L) (median, IQR)	1.00 (0.74–1.37)	1.00 (0.76–1.40)	0.98 (0.72–1.33)
Fasting glucose (mmol/L)	4.77 (0.97)	4.72 (0.64)	4.81 (1.20)
Fasting insulin (pmol/L) (median, IQR)	38.20 (25.70–55.56)	40.28 (27.09–57.64)	36.81 (24.31–54.17)
HOMA-IR (median, IQR)	1.13 (0.74–1.75)	1.18 (0.78–1.78)	1.09 (0.71–1.70)
Framingham risk score (%) (median, IQR)	1.86 (1.02–3.45)	1.90 (1.05–3.44)	1.82 (0.98–3.46)

Values in mean with standard deviation unless otherwise specified

*HDL-C* high density lipoprotein cholesterol, *IQR* interquartile range, *LDL-C* low density lipoprotein cholesterol, *TG* triglycerides; *Total-C* total cholesterol

^a^Data only available for the South African site, n=575 for marital status, *n* = 571 for employment status, *n* = 573 for having children

^b^History or use of medication

^c^Data only available for the South African site, n=574 for current smoking, *n* = 573 for current alcohol use, *n* = 562 for current drug use

**Fig. 1:**
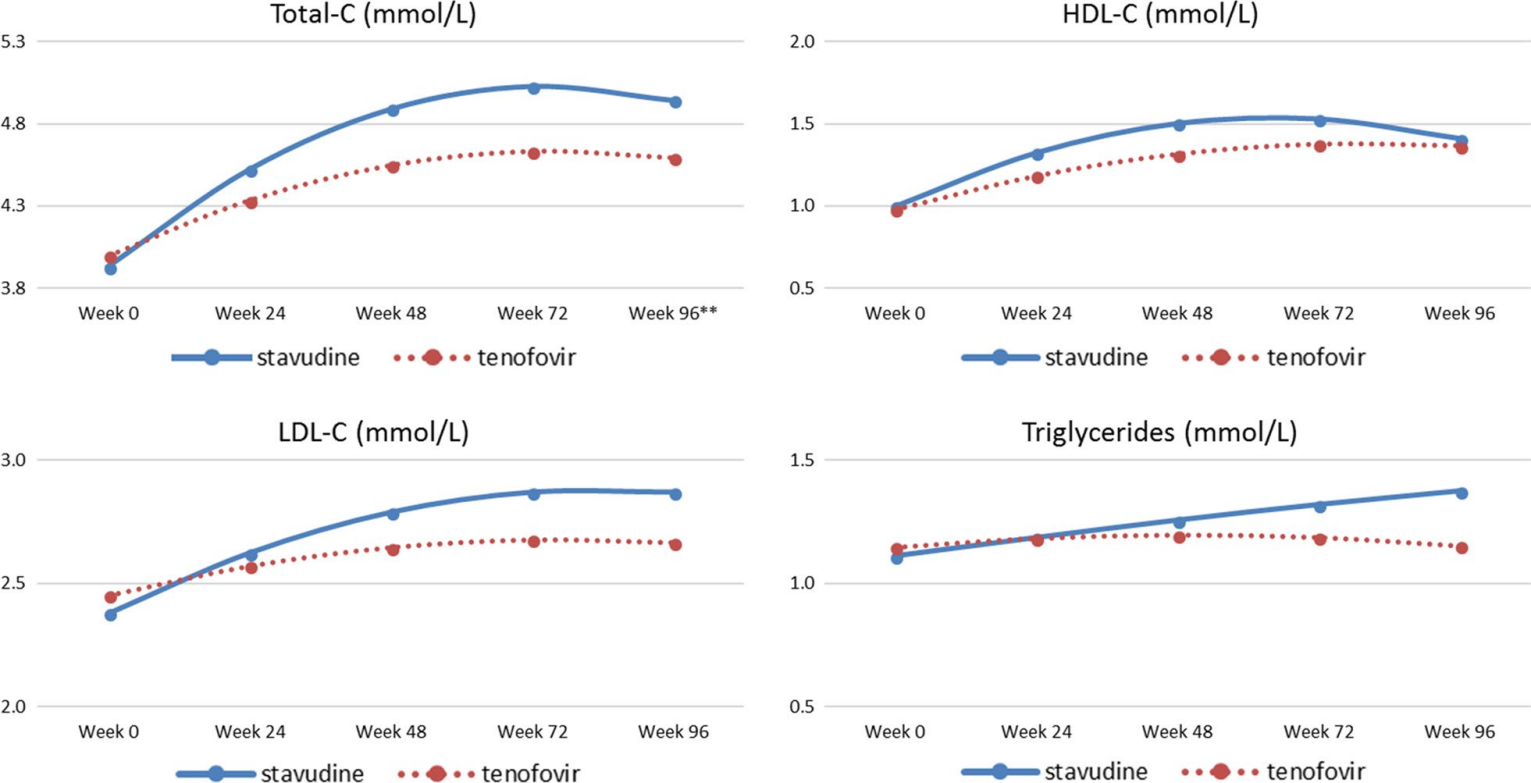
Trends in estimated marginal mean lipid levels in each study group. *HDL-C* high density lipoprotein cholesterol, *LDL-C* low densiy lipoprotein cholesterol, *Total-C* total cholesterol

Figure 1 shows the trend in lipid levels following treatment initiation (Fig. 1). Total-C, HDL-C and LDL-C increased over the course of 96 weeks with a sharper increase in the group receiving stavudine than in the group receiving tenofovir, *p* < 0.001 for Total-C and HDL-C, and *p* = 0.036 for LDL-C (Table 2, see Additional file 2: Table S2). TG levels decreased from week 24 in the group on tenofovir and the estimated mean at week 96 was almost identical to the estimated mean at baseline (1.148 mmol/L (95% CI 1.102–1.197) at baseline versus 1.154 mmol/L (95% CI 1.097–1.212) at week 96).

**Table 2:** Estimated changes per month in lipid levels, insulin resistance and Framingham risk score

Variable	Increase per month Regression coefficient (95% CI)^#^	Increase per month^2^ Regression coefficient (95% CI)^#^	*p*
Total-C (mmol/L)			
Stavudine	0.1275 (0.1035–0.1514)	− 0.0037 (− 0.0047 to − 0.0027)	
Tenofovir	0.0719 (0.0621–0.0818)	− 0.0020 (− 0.0025 to − 0.0016)	< 0.001
HDL-C (mmol/L)			
Stavudine	0.0721 (0.0572–0.0870)	− 0.0024 (− 0.0031 to − 0.0018)	
Tenofovir	0.0433 (0.0372–0.0495)	− 0.0012 (− 0.0014 to − 0.0009)	< 0.001
LDL-C (mmol/L)			
Stavudine	0.0516 (0.0324–0.0709)	− 0.0013 (− 0.0021 to − 0.0005)	
Tenofovir	0.0254 (0.0175–0.0334)	− 0.0007 (− 0.0010 to − 0.0004)	0.036
Log TG (mmol/L)			
Stavudine	0.0125 (− 0.0017–0.0268)	− 0.0001 (− 0.0007–0.0005)	
Tenofovir	0.0073 (0.0014–0.0132)	− 0.0003 (− 0.0006 to − 0.0001)	0.058
Glucose (mmol/L)			
Stavudine	0.0689 (0.0393–0.0980)	− 0.0021 (− 0.0033 to − 0.0009)	
Tenofovir	0.0679 (0.0558–0.0800)	− 0.0020 (− 0.0026 to − 0.0015)	0.991
Insulin (pmol/L)			
Stavudine	0.3941 (− 1.3102–2.0984)	0.0045 (− 0.0659–0.0749)	
Tenofovir	0.0042 (− 0.7001–0.7086)	0.0231 (− 0.0059–0.0521)	0.677
Log HOMA-IR			
Stavudine	0.0242 (0.0006–0.0479)	− 0.0004 (− 0.0014–0.0006)	
Tenofovir	0.0158 (0.0060–0.0255)	− 0.0002 (− 0.0006–0.0003)	0.756
Logit Framingham risk score*			
Stavudine	0.0100 (− 0.0050–0.0251)	− 0.0001 (− 0.0008–0.0005)	
Tenofovir	− 0.0047 (− 0.0109–0.0016)	0.0002 (0.0000–0.0005)	0.144

*CI* confidence interval, *HDL-C* high density lipoprotein cholesterol, *LDL-C* low density lipoprotein cholesterol, *OR* odds ratio, *TG* triglycerides, *Total-C* total cholesterol

*Data for South African site only

^#^The model includes adjustment for age, gender, site, body mass index and viral load

Month^2^, time was used as a quadratic term to take non-linearity into account. *p* values were estimated with a likelihood ratio test for both interactions. See “Methods” for details

**Fig. 2:**
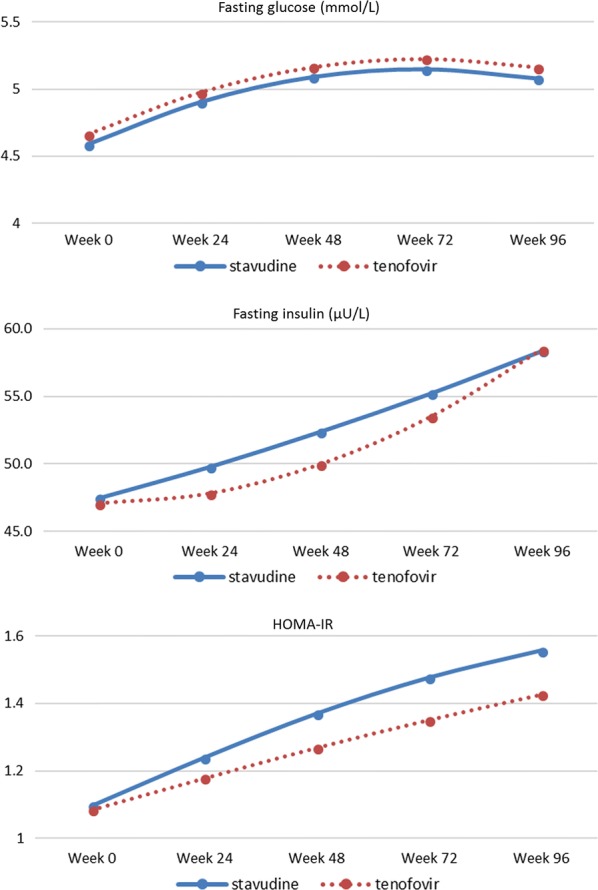
Trends in estimated marginal mean levels of indicators of insulin resistance

Glucose values were similar in both groups, with a steep increase in the first 48 weeks (from 4.63 mmol/L (95% CI 4.56–4.69) at baseline to 5.13 mmol/L (95% CI 5.06–5.20) at week 48) and values tended to level off from week 48 to reach 5.12 mmol/L (95% CI 5.05–5.31) at week 96 (see Fig. 2 for trend per treatment arm and Additional file 2: Table S2). Insulin increased over 96 weeks, with no difference detected between arms (*p* = 0.677, Table 2). The increase in insulin resistance per month (with HOMA-IR) was not different for participants on stavudine versus tenofovir (*p* = 0.698; Table 2).

**Fig. 3:**
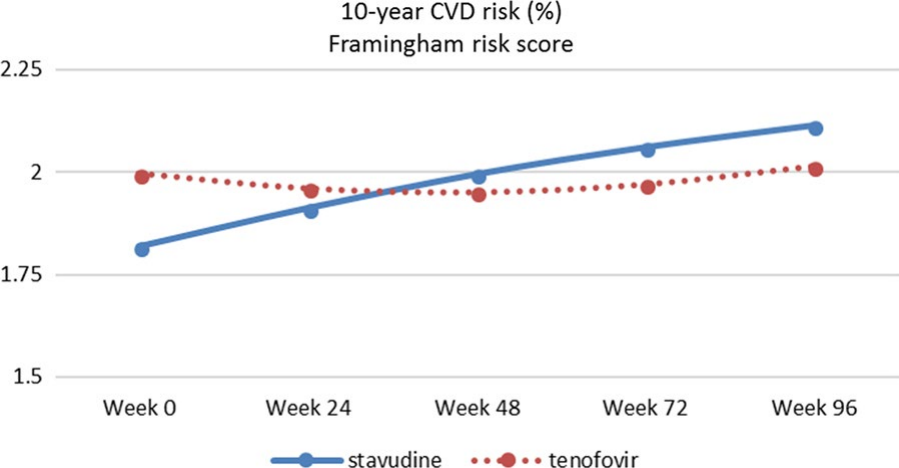
Trend in estimated marginal mean levels of 10-year CVD risk

Framingham risk score rose from 1.90% (1.84–1.98%) at baseline to 2.06 (1.98–2.15%) at week 96 for the total group (see Fig. 3 for trend per treatment arm and Additional file 2: Table S2). FRS in the stavudine group was significantly lower at baseline than in the tenofovir group, 1.82% (95% CI 1.73–1.92) and 2.00% (95% CI 1.89–2.10) respectively, *p* = 0.013. The risk in the group on stavudine went up to 2.11% (95% CI 1.99–2.24) at week 96. The risk in the group on tenofovir decreased initially to 1.94% (95% CI 1.85–2.05) at week 48 to increase to 2.01% (95% CI 1.90–2.13) at week 96. The overall trend between the groups was not significantly different, *p* = 0.144.

Age, sex and BMI were associated with all lipid subcomponents, glucose, insulin, HOMA-IR and FRS (Additional file 3: Table S3a–h). The 10-year CVD risk according to the FRS was almost double for males compared to females at week 96, 2.88% (95% CI 2.68–3.00) for males versus 1.50% (95% CI 1.42–1.58) for females, *p* < 0.001. Log viral load at baseline was associated with all lipid subcomponents (with a higher log viral load linked to a lower Total-C, HDL-C and LDL-C) but not to glucose, insulin, HOMA-IR or FRS. Indian participants had a worse lipid profile and glucose homeostasis compared to their African counterparts. For example, at baseline Total-C was 4.38 mmol/L (95% CI 4.21–4.55) for the Indian site and 3.72 mmol/L (95% CI 3.66–3.79) for the South African site, and fasting glucose was 5.05 mmol/L (95% CI 4.90–5.22) for the Indian site versus 4.45 mmol/L (95% CI 4.38–4.51) for the South African site, *p* < 0.001 for both comparisons. In addition to these differences in baseline levels, Indian participants had a sharper increase in total-C, LDL-C and TG, and a larger decrease in HDL-C with treatment (See Additional file 3: Table S3a–d). The effect of stavudine and tenofovir on lipid components, as described above, was similar for all three populations (South Africa, Uganda and India).

## Discussion

Initiation of low-dose stavudine and tenofovir with a backbone of lamivudine and efavirenz resulted in a rise in lipid levels with a worse lipid profile for stavudine compared to tenofovir. Insulin resistance went up but there was no difference in trend between both groups. CVD risk was low in general and with no statistically significant difference between the groups over time.

Untreated HIV infection is characterized by an increase in TG and a decrease in both LDL-C and HDL-C [8, 21, 22]. The disturbance in lipid levels is likely a result of chronic inflammation that accompanies HIV infection [23]. Chronic inflammation influences the way lipids are metabolized and activated. Oxidized LDL and HDL may directly induce monocyte and endothelial cell activation, and this is related to the development of CVD [24].

Studies on initiation of ART have attributed both improvement, as well as deterioration, in lipid levels to ART [8, 22, 24–26]. A meta-analysis of cardiovascular risk factors in relation to HIV and ART in sub-Saharan Africa found that ART was associated with an increase in HDL-C and a decrease in TG, but also with an increase in LDL-C [2]. Liu et al. investigated changes in lipid levels during 3 years following ART initiation in Tanzania. They found an increase in LDL and HDL, and a decrease in TGs in the first 6 months. Between 6 months and 3 years HDL levels topped off and TG levels continued to increase [25]. Our results are broadly in line with these findings. We did not see a decrease in TG between treatment initiation and week 96, but the TG level in the group receiving tenofovir at week 96 was approximately the same as the level before treatment initiation. Our results underline the importance of taking treatment duration into account.

There is no agreed threshold to define insulin resistance in an urban African population [27] but based on a study in an urban Ghanaian population, a HOMA-IR cut-off of 2.3 seems appropriate [28]. According to this definition, neither low-dose stavudine nor tenofovir resulted in significant insulin resistance. Stavudine’s mitochondrial toxicity is likely dose dependent [29, 30], and the low dose used in this study may account for the lack of impact on glucose metabolism. We can, however, not exclude the possibility that the drug’s deleterious effects could still occur with more extended durations of treatment.

Lipid levels and insulin resistance are increased in both treatment arms. This may partly rely on the effects of efavirenz, as this drug is known to alter lipid levels and glucose metabolism [11, 15]. Our findings underline the need for more ‘lipid-friendly’ ART, of which dolutegravir is a promising alternative [31].

CVD risk according to the 10-year FRS increased in the total group with 0.16% between baseline and week 96. This was statistically significant, but it’s questionable whether this slight increase over almost 2 years is clinically relevant. There was a trend towards a sharper increase in CVD risk in the group receiving stavudine, most likely reflecting the steeper increase in LDL-C and TG in this group compared to the group on tenofovir. At the same time, it would be important to consider the relative contribution of ART to well-known modifiable cardiovascular risk factors such as obesity, smoking, diet, and physical inactivity.

Indian participants had substantially worse lipid profiles and glucose homeostasis compared to the African participants. This is a well-known phenomenon that is likely dependent on genetic factors and lifestyle factors such as being underweight during infancy, diet and physical activity [32–34].

Strengths of the study are the large, representative sample including participants from three countries, the randomized design, the standardized and regular assessment of lipids, glucose and insulin, and the follow up duration of almost 2 years. However, some limitations need to be recognized. Information on socio-demographics, alcohol use, drug use and smoking was only collected for the South African site, relied on patient reporting as opposed to biochemical measures, and only at baseline. No information on family history of CVD was collected, so we could not quantify CVD risk with other CVD risk prediction scores such as the Data collection on Adverse events of Anti-HIV Drugs (D:A:D) score, which is considered to be a more accurate risk prediction tool for HIV-infected populations than the FRS [35]. Also, we could not examine effects of the metabolic syndrome as waist circumference had not been measured. In line with an intention-to-treat approach for clinical trials, we only corrected for viral load at baseline, even though viral load at follow-up was available in the data. Both adherence and the proportion of participants with suppressed viral load between treatment groups were approximately equal. We therefore assume that any bias is small. Finally, the study did not include an HIV-negative control group to compare our findings to age-related, HIV-unrelated changes in lipid levels and insulin resistance over time.

## Conclusion

This study showed that low-dose stavudine has more deleterious effects on lipids than tenofovir. However, the impact of stavudine on this short term appears to be small, and it’s questionable whether the increase in lipid levels is clinical relevant in this relatively young population with a low CVD risk. The need to monitor lipid and glucose levels has to be determined using CVD risk calculators that take other risk factors like age and hypertension into account. There should be extra awareness for CVD risk prevention in the Indian population. We recommend to measure lipids and glucose at the earliest a year after treatment initiation, as levels change substantially following treatment initiation.

## Additional files

**Additional file 1: Table S1.** Title: Laboratory methods. Description of data: this table includes details on the measurement methods of lipids, glucose, insulin, HIV viral load and CD4+ cell count.

**Additional file 2: Table S2.** Title: Estimated means with 95% confidence intervals per outcome. Description of data: This table presents estimates of the means for each treatment group at 4 follow-up measurements based on a linear mixed model with correction for age, sex, site of inclusion (South-Africa, Uganda or India), body mass index and viral load at baseline (see “Methods” for details).

**Additional file 3: Table S3.** Title: Generalized linear mixed models. Description of data: generalized linear mixed models for the outcomes total cholesterol, HDL cholesterol, LDL cholesterol, triglycerides, glucose, insulin, HOMA-IR and Framingham risk score.
