# Supplementary material for: Lipid levels, insulin resistance and cardiovascular risk over 96 weeks of antiretroviral therapy: a randomised controlled trial comparing low-dose stavudine and tenofovir
Source: Retrovirology. 2018 Dec 14;15:77. doi: 10.1186/s12977-018-0460-z (PMC6295103; doi:10.1186/s12977-018-0460-z)
Supplement: Supplementary file 1 — Additional file 1: Table S1. Title: Laboratory methods. Description of data: this table includes details on the measurement methods of lipids, glucose, insulin, HIV viral load and CD4+ cell count. [file 12977_2018_460_MOESM1_ESM.docx]

| **Laboratory methods** |  |
| --- | --- |
| Total cholesterol, HDL-C, TG, glucose | Cobas Integra 400 autoanalyzer (Roche Diagnostics Ltd., Indianapolis, Ind., USA) |
| LDL-C | Calculated using the Friedman formula: LDL-C = Total-C – HDL-C – (0.45 * TG) |
| Insulin | ABBOTT ARCHITECT |
| CD4 cell count | EPICS XL/MCL Analyzer Cytomics FC500 Flow Cytometer MPL/Cellmek (Beckman Coulter Inc. California, USA) |
| HIV viral load | Roche Cobas Amplicor/Cobas AmpliPrep/Cobas Taqman/Easymag/EasyQ Analyser |
| HDL-C, high density lipoprotein cholesterol; LDL-C, low density lipoprotein cholesterol; TG, triglycerides. | |

**Supplementary table 1. Laboratory methods**
