# Supplementary material for: Lipid levels, insulin resistance and cardiovascular risk over 96 weeks of antiretroviral therapy: a randomised controlled trial comparing low-dose stavudine and tenofovir
Source: Retrovirology. 2018 Dec 14;15:77. doi: 10.1186/s12977-018-0460-z (PMC6295103; doi:10.1186/s12977-018-0460-z)
Supplement: Supplementary file 2 — Additional file 2: Table S2. Title: Estimated means with 95% confidence intervals per outcome. Description of data: This table presents estimates of the means for each treatment group at 4 follow-up measurements based on a linear mixed model with correction for age, sex, site of inclusion (South-Africa, Uganda or India), body mass index and viral load at baseline (see “Methods” for details). [file 12977_2018_460_MOESM2_ESM.docx]

**Supplementary table 2. Estimated means with 95% confidence intervals per outcome**

|  | **Baseline** | **Week 24** | **Week 48** | **Week 72** | **Week 96** |
| --- | --- | --- | --- | --- | --- |
| Total-C (mmol/L) |  |  |  |  |  |
| stavudine | 3.93  (3.85 – 4.02) | 4.54  (4.44 – 4.61) | 4.89  (4.80 – 4.98) | 5.03  (4.94 – 5.12) | 4.94  (4.84 – 5.05) |
| tenofovir | 4.00  (3.92 – 4.08) | 4.33  (4.25 – 4.42) | 4.55  (4.46 – 4.63) | 4.63  (4.54 – 4.72) | 4.59  (4.49 – 4.69) |
| HDL-C (mmol/L) |  |  |  |  |  |
| stavudine | 1.00  0.96 – 1.04) | 1.32  (1.29 – 1.36) | 1.50  (1.46 – 1.54) | 1.53  (1.49 – 1.57) | 1.41  (1.36 – 1.46) |
| tenofovir | 0.98  (0.94 – 1.02) | 1.18  (1.15 – 1.22) | 1.32  (1.28 – 1.36) | 1.38  (1.33 – 1.42) | 1.37  (1.32 – 1.41) |
| LDL-C (mmol/L) |  |  |  |  |  |
| stavudine | 2.38  (2.31 – 2.45) | 2.62  (2.55 – 2.70) | 2.79  (2.71 – 2.87) | 2.87  (2.78 – 2.95) | 2.87  (2.78 – 2.96) |
| tenofovir | 2.45  (2.38 – 2.52) | 2.57  (2.50 – 2.64) | 2.65  (2.57 – 2.72) | 2.68  (2.59 – 2.76) | 2.67  (2.57 – 2.76) |
| TG (mmol/L) |  |  |  |  |  |
| stavudine | 1.11  (1.06 – 1.16) | 1.18  (1.14 – 1.23) | 1.26  (1.20 – 1.31) | 1.32  (1.26 – 1.38) | 1.37  (1.31 – 1.45) |
| tenofovir | 1.15  (1.10 – 1.20) | 0.18  (1.14 – 1.23) | 1.20  (1.15 – 1.25) | 1.19  (1.14 – 1.24) | 1.15  (1.10 – 1.21) |
| Glucose (mmol/L) |  |  |  |  |  |
| stavudine | 4.59  (4.50 – 4.67) | 4.90  (4.82 – 4.98) | 5.09  (5.00 – 5.12) | 5.15  (5.06 – 5.23) | 5.08  (4.98 – 5.18) |
| tenofovir | 4.66  (4.58 – 4.75) | 4.98  (4.90 – 5.05) | 5.16  (5.08- 5.25) | 5.23  (5.14 – 5.31) | 5.16  (5.07 – 5.26) |
| Insulin (µmol/L) |  |  |  |  |  |
| stavudine | 47.48  (44.26 – 50.71) | 49.80  (47.08 – 52.52) | 52.40  (48.71 – 56.09) | 55.24  (50.82 – 59.66) | 58.37  (52.43 – 64.32) |
| tenofovir | 47.07  (43.88 – 50.27) | 47.80  (45.14 – 50.46) | 49.96  (46.33 – 53.59) | 53.49  (49.17 – 57.81) | 58.45  (52.71 – 64.19) |
| HOMA-IR |  |  |  |  |  |
| stavudine | 1.10  (1.03 – 1.16) | 1.24  (1.18 – 1.30) | 1.37  (1.29 – 1.45) | 1.48  (1.39 – 1.56) | 1.56  (1.46 – 1.66) |
| tenofovir | 1.09  (1.02 – 1.15) | 1.18  (1.12 – 1.24) | 1.27  (1.20 – 1.34) | 1.35  (1.28 – 1.43) | 1.43  (1.34 – 1.52) |
| Framingham risk score |  |  |  |  |  |
| stavudine | 1.82  (1.73 – 1.92) | 1.91  (1.82 – 2.01) | 1.99  (1.89 – 2.10) | 2.06  (1.96 – 1.27) | 2.11  (1.99 – 2.24) |
| tenofovir | 2.00  (1.89 – 2.10) | 1.96  (1.87 – 2.06) | 1.95  (1.85 – 2.05) | 1.97  (1.87 – 2.07) | 2.01  (1.90 – 2.13) |
| Total-C: total cholesterol, HDL-C: high density lipoprotein cholesterol, LDL-C: low density lipoprotein cholesterol, TG: triglycerides | | | | | |

Note: This table presents estimates of the means for each treatment group at 4 follow-up measurements based on a linear mixed model with correction for age, sex, site of inclusion (South-Africa, Uganda or India), body mass index and viral load at baseline (see methods for details).
