# Supplementary material for: Lipid levels, insulin resistance and cardiovascular risk over 96 weeks of antiretroviral therapy: a randomised controlled trial comparing low-dose stavudine and tenofovir
Source: Retrovirology. 2018 Dec 14;15:77. doi: 10.1186/s12977-018-0460-z (PMC6295103; doi:10.1186/s12977-018-0460-z)
Supplement: Supplementary file 3 — Additional file 3: Table S3. Title: Generalized linear mixed models. Description of data: generalized linear mixed models for the outcomes total cholesterol, HDL cholesterol, LDL cholesterol, triglycerides, glucose, insulin, HOMA-IR and Framingham risk score. [file 12977_2018_460_MOESM3_ESM.docx]

**Supplementary Tables 3a – 3h.** Linear mixed models

| **Supplementary Table 3a: Total-C (mmol/L)** | | |
| --- | --- | --- |
|  | **Regression coefficient (95% CI)** | ***P*** |
| Intercept | 3.0826 (2.5934 – 3.5719) | < 0.001 |
| Stavudine | -0.0670 (-0.1668 – 0.0328) | 0.188 |
| Tenofovir | REF |  |
| Time per month | 0.0719 (0.0621 – 0.0818) | < 0.001 |
| Stavudine * time | 0.0555 (0.0415 – 0.0696) | < 0.001 |
| Tenofovir * time | REF |  |
| Time per month^2^ | -0.0020 (-0.0025 - -0.0016) | < 0.001 |
| Time per month^2^ * stavudine | -0.0017 (-0.0022 - -0.0011) | < 0.001 |
| Time per month^2^ * tenofovir | REF |  |
| Age (years) | 0.0206 (0.0148 – 0.0264) | < 0.001 |
| Sex Male | -0.1403 (-0.2403 - -0.0402) | 0.006 |
| Female | REF |  |
| Site South Africa | REF |  |
| Uganda | 0.0749 (-0.0270 – 0.1769) | 0.150 |
| India | 0.6572 (0.4769 – 0.8377) | < 0.001 |
| BMI (kg/m^2^) | 0.0333 (0.0216 – 0.0451) | < 0.001 |
| Log viral load (copies/mL) | -0.1578 (-0.2267 - -0.0889) | < 0.001 |

| **Supplementary Table 3b: HDL-C (mmol/L)** | | |
| --- | --- | --- |
|  | **Regression coefficient (95% CI)** | ***P*** |
| Intercept | 1.5426 (1.3234 – 1.7618) | < 0.001 |
| Stavudine | 0.0208 (-0.0296 – 0.0712) | 0.419 |
| Tenofovir | REF |  |
| Time per month | 0.0433 (0.0372 – 0.0495) | < 0.001 |
| Stavudine * time | 0.0287 (0.0200 – 0.0375) | < 0.001 |
| Tenofovir * time | REF |  |
| Time per month^2^ | -0.0012 (-0.0014 - -0.0009) | < 0.001 |
| Time per month^2^ * stavudine | -0.0013 (-0.0016 - -0.0009) | < 0.001 |
| Time per month^2^ * tenofovir | REF |  |
| Age (years) | 0.0040 (0.0014 – 0.0065) | 0.003 |
| Sex Male | -0.1266 (-0.1712 - -0.0819) | < 0.001 |
| Female | REF |  |
| Site South Africa | REF |  |
| Uganda | -0.0994 (-0.1449 – 0.0540) | < 0.001 |
| India | -0.2550 (0.3357 – 0.1742) | < 0.001 |
| BMI (kg/m^2^) | -0.0064 (-0.0116 – -0.0013) | 0.017 |
| Log viral load (copies/mL) | -0.0762 (-0.1070 - -0.0454) | < 0.001 |

| **Supplementary Table 3c: LDL-C (mmol/L)** | | |
| --- | --- | --- |
|  | **Regression coefficient (95% CI)** | ***P*** |
| Intercept | 1.6638 (1.2303 – 2.0973) | < 0.001 |
| Stavudine | -0.0714 (-0.1584 – 0.0157) | 0.108 |
| Tenofovir | REF |  |
| Time per month | 0.0254 (0.0175 – 0.0334) | < 0.001 |
| Stavudine * time | 0.0262 (0.0149 – 0.0375) | < 0.001 |
| Tenofovir * time | REF |  |
| Time per month^2^ | -0.0007 (-0.0010 - -0.0004) | < 0.001 |
| Time per month^2^ * stavudine | -0.0006 (-0.0011 - -0.0001) | 0.010 |
| Time per month^2^ * tenofovir | REF |  |
| Age (years) | 0.0136 (0.0085 – 0.0188) | < 0.001 |
| Sex Male | -0.0990 (-0.188 - -0.0103) | 0.029 |
| Female | REF |  |
| Site South Africa | REF |  |
| Uganda | 0.1012 (0.0108 – 0.1916) | 0.028 |
| India | 0.6367 (0.4768 – 0.7965) | < 0.001 |
| BMI (kg/m^2^) | -0.0278 (0.0174 – 0.0382) | < 0.001 |
| Log viral load (copies/mL) | -0.1117 (-0.1728 - -0.0507) | < 0.001 |

| **Supplementary Table 3d: log TG (mmol/L)** | | |
| --- | --- | --- |
|  | **Regression coefficient (95% CI)** | ***P*** |
| Intercept | -1.3038 (-1.5338 – -1.0738) | < 0.001 |
| Stavudine | -0.0331 (-0.0838 – 0.0175) | 0.199 |
| Tenofovir | REF |  |
| Time per month | 0.0254 (0.0175 – 0.0334) | < 0.001 |
| Stavudine * time | 0.0262 (0.0149 – 0.0375) | < 0.001 |
| Tenofovir * time | REF |  |
| Time per month^2^ | 0.0073 (0.0014 - 0.0131) | 0.016 |
| Time per month^2^ * stavudine | 0.0053 (-0.0031 - -0.0136) | 0.217 |
| Time per month^2^ * tenofovir | REF |  |
| Age (years) | 0.0055 (0.0028 – 0.0082) | < 0.001 |
| Sex Male | 0.1530 (0.1061 - 0.1999) | 0.029 |
| Female | REF |  |
| Site South Africa | REF |  |
| Uganda | 0.1358 (0.0880 – 0.1836) | < 0.001 |
| India | 0.5651 (0.4803 – 0.6499) | < 0.001 |
| BMI (kg/m^2^) | 0.0215 (0.0160 – 0.0270) | < 0.001 |
| Log viral load (copies/mL) | 0.0883 (0.0560 – 0.1206) | < 0.001 |

| **Supplementary Table 3e: glucose (mmol/L)** | | |
| --- | --- | --- |
|  | **Regression coefficient (95% CI)** | ***P*** |
| Intercept | 3.4479 (2.9766 – 3.9190) | < 0.001 |
| Stavudine | -0.0758 (-0.1823 – 0.0308) | 0.163 |
| Tenofovir | REF |  |
| Time per month | 0.0679 (0.0558 – 0.0800) | < 0.001 |
| Stavudine * time | 0.0007 (-0.0165 – 0.0179) | 0.933 |
| Tenofovir * time | REF |  |
| Time per month^2^ | -0.0021 (-0.0026 - -0.0015) | < 0.001 |
| Time per month^2^ * stavudine | -0.0001 (-0.0078 - 0.0007) | 0.891 |
| Time per month^2^ * tenofovir | REF |  |
| Age (years) | 0.0093 (0.0038 – 0.0149) | 0.001 |
| Sex Male | 0.1530 (0.1061 - 0.1999) | 0.029 |
| Female | REF |  |
| Site South Africa | REF |  |
| Uganda | -0.0756 (-0.1733 – 0.0221) | 0.129 |
| India | 0.6150 (0.4413 – 0.7887) | < 0.001 |
| BMI (kg/m^2^) | 0.0245 (0.0132 – 0.0357) | < 0.001 |
| Log viral load (copies/mL) | 0.0016 (-0.0645 – 0.0678) | 0.961 |

| **Supplementary Table 3f: insulin (pmol/L)** | | |
| --- | --- | --- |
|  | **Regression coefficient (95% CI)** | ***P*** |
| Intercept | 10.4996 (-4.5763 – 25.5756) | 0.172 |
| Stavudine | 0.4126 (-3.6727 – 4.4979) | 0.843 |
| Tenofovir | REF |  |
| Time per month | 0.0042 (-0.7001 – 0.7086) | 0.991 |
| Stavudine * time | 0.3898 (-0.6101 – 1.3898) | 0.445 |
| Tenofovir * time | REF |  |
| Time per month^2^ | 0.0231 (-0.0059 – 0.0521) | 0.118 |
| Time per month^2^ * stavudine | -0.0187 (-0.0601 – 0.0223) | 0.891 |
| Time per month^2^ * tenofovir | REF |  |
| Age (years) | -0.5081 (-0.6839 – -0.3325) | < 0.001 |
| Sex Male | -5.4980 (-8.5468 – -2.4493) | < 0.001 |
| Female | REF |  |
| Site South Africa | REF |  |
| Uganda | -4.736 (-7.8346 – -1.6378) | 0.003 |
| India | 19.166 (13.6368 – 24.6955) | < 0.001 |
| BMI (kg/m^2^) | 2.2421 (1.8834 – 2.6010) | < 0.001 |
| Log viral load (copies/mL) | -0.0724 (-2.1758 – 2.0310) | 0.946 |

| **Supplementary Table 3g: log HOMA-IR** | | |
| --- | --- | --- |
| **Log HOMA-IR** | **Regression coefficient (95% CI)** | ***P*** |
| Intercept | -0.8821 (-1.1714 – -0.5928) | < 0.001 |
| Stavudine | 0.0098 (-0.0635 – 0.0832) | 0.793 |
| Tenofovir | REF |  |
| Time per month | 0.0158 (0.0060 – 0.0255) | 0.002 |
| Stavudine * time | 0.0085 (-0.0054 – 0.0223) | 0.231 |
| Tenofovir * time | REF |  |
| Time per month^2^ | -0.0002 (-0.0006 – 0.0003) | 0.465 |
| Time per month^2^ * stavudine | -0.0002 (-0.0008 – 0.0004) | 0.455 |
| Time per month^2^ * tenofovir | REF |  |
| Age (years) | -0.0097 (-0.0131 – -0.0063) | < 0.001 |
| Sex Male | -0.0945 (-0.1531 – -0.0359) | 0.002 |
| Female | REF |  |
| Site South Africa | REF |  |
| Uganda | -0.1839 (-0.2435 – -0.1243) | < 0.001 |
| India | 0.4052 (0.2986 – 0.5117) | < 0.001 |
| BMI (kg/m^2^) | 0.0519 (0.0450 – 0.0588) | < 0.001 |
| Log viral load (copies/mL) | 0.0119(-0.0286 – 0.0523) | 0.565 |

| **Supplementary Table 3h: logit Framingham risk score** | | |
| --- | --- | --- |
|  | **Regression coefficient (95% CI)** | ***P*** |
| Intercept | -7.7377 (-8.0744 – -7.4009) | < 0.001 |
| Stavudine | -0.0949 (-0.1697 – -0.0201) | 0.013 |
| Tenofovir | REF |  |
| Time per month | -0.0047 (-0.0109 – 0.0016) | 0.141 |
| Stavudine * time | 0.0147 (0.0059 – 0.0236) | 0.001 |
| Tenofovir * time | REF |  |
| Time per month^2^ | 0.0002 (-0.0000 – 0.0005) | 0.083 |
| Time per month^2^ * stavudine | -0.0004 (-0.0007 – -0.0000) | 0.049 |
| Time per month^2^ * tenofovir | REF |  |
| Age (years) | 0.0810 (0.0765 – 0.0855) | < 0.001 |
| Sex Male | 0.6516 (0.5791 – 0.7241) | < 0.001 |
| Female | REF |  |
| BMI (kg/m^2^) | 0.0278 (0.0194 – 0.0362) | < 0.001 |
| Log viral load (copies/mL) | 0.0049 (-0.0419 – 0.0516) | 0.839 |
